# Editorial: Genetic factors in male infertility

**DOI:** 10.3389/fgene.2023.1187445

**Published:** 2023-04-05

**Authors:** Xiaojin He, Shenmin Yang, Mingrong Lv

**Affiliations:** ^1^ Reproductive Medicine Center, Department of Obstetrics and Gynecology, The First Affiliated Hospital of Anhui Medical University, Hefei, China; ^2^ Center for Reproduction and Genetics, Suzhou Municipal Hospital Suzhou, Gusu School, The Affiliated Suzhou Hospital of Nanjing Medical University, Nanjing Medical University, Suzhou, China

**Keywords:** male infertility, teratozoospermia, azoospermia, genetic factors, gene viriants

Our Research Topic is “Genetic Factors in Male Infertility” and the goal of this Research Topic is to collect more papers on the aspect of identifying different gene variants and various types of RNAs mainly focused on male infertility including azoospermia, oligozoospermia, teratozoospermia, and asthenozoospermia. In order to achieve this goal, we carefully reviewed every submitted manuscript and screened for highly qualified reviewers. Eventually, we accepted and published seven articles including four “Original Research” articles, one “Brief Research Report” article, one “Case Report” article, and one “Review”. These articles covered the genetic factors of non-obstructive azoospermia and teratozoospermia and the clinical outcomes of men with non-mosaic Klinefelter syndrome (KS). Xie et.al identify a homozygous missense variant in *DND1* that causes non-obstructive azoospermia in humans (Xie et.al). Aprea et.al. identify the pathogenic gene variants in *CCDC39*, *CCDC40*, *RSPH1*, *RSPH9*, *HYDIN,* and *SPEF2* that cause defects of sperm flagella composition and male infertility. They find that immunofluorescence microscopy in sperm cells is a valuable tool to identify flagellar defects related to the axonemal ruler, radial spoke head, and the central pair apparatus (Aprea et.al.). Yuan et al. finds a Gly684Ala substitution in the androgen receptor that causes azoospermia (Yuan et al.) and Fang et al. reports the phenotypic findings and genetic considerations of congenital absence of the vas deferens with hypospadias or without hypospadias: (Fang et al.), which might advance the genetic diagnosis and clinical genetic counseling for male infertility. Zhu et al. also verifies that FISH could analyze numerical chromosomal abnormalities in the sperm of Robertsonian translocation der (13; 14) (q10; q10) carriers (Zhu et al.). Moreover, Wang et.al review various phenotypes resulting from different pathogenic genes, including sperm ultrastructure and encoding proteins with their location and functions as well as assisted reproductive technology outcomes, providing additional clinical views and broadening the understanding of this disease (Wang et.al). Finally, Xu et al. reports a case of the birth of a boy after intracytoplasmic sperm injection using ejaculated spermatozoa from a non-mosaic KS man with normal sperm motility, which increases our knowledge of non-mosaic KS (Xu et al.). The findings in these studies broaden our understanding of genetic factors in male infertility and were also included in our Research Topic scope.

